# Keeping them distant for ultimate brightness

**DOI:** 10.1038/s41377-025-01760-x

**Published:** 2025-02-13

**Authors:** Maximilian Stremel, Markus Suta

**Affiliations:** Inorganic Photoactive Materials, Faculty of Mathematics and Natural Sciences, Heinrich Heine University Düsseldorf, Düsseldorf, Germany

**Keywords:** Inorganic LEDs, Fluorescence spectroscopy

## Abstract

While strong absorption is best achieved with high activator concentrations, luminescence brightness typically suffers in that case due to energy migration and concentration quenching. The teams around Xia and Zhong have reported about an aphthitalite-type Eu-activated phosphate that breaks with this paradigm and enables the sustainment of high photoluminescence quantum yields (>40%) up to concentrations as high as 70 mol% Eu. Mixing of that phosphor with a low (0.8%) and high (70%) Eu fraction results in almost natural white light with excellent color rendition and correlated color temperature that could serve as a new instructive concept for next-generation phosphor-converted white LEDs.

## Abstract (web summary)

A clever structural concept in Eu^2+^-activated glaserite-type Rb_3_Y(PO_4_)_2_ breaks with a fundamental paradigm of phosphor design and allows keeping the internal quantum yield high despite high activator concentrations. Mixing that phosphate with a low (0.8 mol%) and high (70 mol%) Eu content paves the way for sustainable warm-white LEDs.

## Graphical abstract

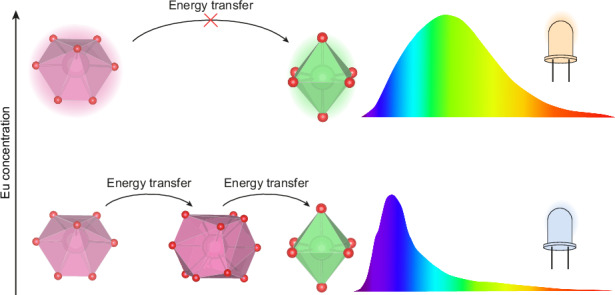

Phosphor-converted white light-emitting diodes (pc-wLEDs) comprise an energy-efficient way for white lighting since the advent of the blue In_1-*x*_Ga_*x*_N LED. Its major breakthrough in the 1990s was honored by the Nobel Prize in Physics in 2014 awarded to the developers Shuji Nakamura, Isamu Akasaki, and Hiroshi Amano^1–4^. Shortly after the commercial success of the blue LED, the company Nichia (at which Shuji Nakamura had also worked at that time) realized that efficient white lighting could be possible by mixing the blue light of the LED chip with emitted yellow light from a ceramic phosphor, Y_3_Al_5_O_12_:Ce^3+^ (YAG:Ce)^5^. That phosphor was originally developed in 1968 by George Blasse and Alfred Bril at Philips Lighting Industries for new cathode ray tubes for emerging color television at that time^6^. Its discoverers would have probably never imagined such an important role in the future of this inorganic phosphor.

Albeit an ingeniously simple way to generate white light, the lack of the cyan and red portion of the visible spectrum makes that two-color concept-based white light appear cold-white with a low color rendering index *R*_a_ and comparably high correlated color temperature (CCT). The CCT is connected to the temperature of a black body radiator giving rise to that emission color and is known from heating a piece of iron, for example. Upon heating, it first becomes orange-red (low CCT) before turning bluish-white (high CCT) at very high temperatures as the maximum of the corresponding Planck spectrum moves towards the visible range by means of Wien’s displacement law (*λ*_max_*T* = const.). Nowadays, more natural and generally appealing white light is thus generated by exciting a green- and red-emitting phosphor with the blue light of the InGaN LED. This three-color concept offers more flexibility in the color space and usually yields more warm-white light with higher color rendition and CCTs that are closer to the CCT of natural sunlight during daytime (~ 5700 K). However, especially the red-emitting phosphor needs to fulfill several practical demands for that purpose. While our eyes are most sensitive to green light during daytime (the spectral range of the peak of the Planck spectrum emitted by the sun’s surface) based on evolutionary reasons, they become insensitive above 650 nm. Thus, a red-emitting phosphor for warm-white lighting should not only be bright, but also show sufficiently narrow emission bands to ensure that there is minimum leakage of light in part of the spectrum the human eye is insensitive to. This would otherwise result in wasted electric power consumption overall lowering the so-called luminous efficacy (in lumens per Watt, lm/W) of a pc-wLED. Nowadays, especially Eu^2+^ with a broad-banded 4f^6^5d^1^ → 4f^7^-based emission is used as an activator for that purpose. The emission color can be tuned all over the visible range depending on the choice of the ligand field. As host compounds, particularly oxido- and nitridosilicates and -aluminates with highly condensed structures, especially of the UCr_4_C_4_ structure type have now been established and commercialized^7–11^ and result in sufficiently narrow emission bands characterized by high thermal quenching temperatures (> 150 °C) and high internal quantum yields.

Despite the huge progress in this area, the currently exploited three-color concept requires two different phosphors. Although silicates and -aluminates are chemically rather stable, it is not readily clear up to now if there will be chemical reactions of the two-phosphor mixture or ion migration processes under long-time working conditions that could potentially degrade the phosphor mixture and thus, diminish the lifetime of the LED. In a recent publication in *Light: Science & Applications*, the teams around Xia and Zhang have now presented an appealingly simple alternative approach that works with the same compound but different activator fractions of Eu^2+^ to achieve warm-white light with excellent color rendition (*R*_a_ = 96.0) and a CCT (= 5393 K) close to the temperature of the sun’s surface (*T* = 5700 K)^12^. For that purpose, they investigated the structural and luminescence properties of the aphthitalite (formerly known as glaserite, K_3_Na(SO_4_)_2_)-type Eu-activated phosphate Rb_3_Y(PO_4_)_2_. Aphthitalite crystallizes in a densely packed structure that can be regarded as a superstructure of that of simple β-K_2_SO_4_^13^. In the structure of Rb_3_Y(PO_4_)_2_, two crystallographically independent Rb sites (12- and (7 + 3)-fold coordinated) and a Y site (6-fold coordinated) are available for occupation by incorporated Eu^2+^ or Eu^3+^ ions. As they figured out by X-ray absorption near the edge and extended fine structure spectroscopy (XANES and EXAFS) at the Eu *L*_3_ edge, low activator fractions lead to major incorporation of Eu^3+^ ions at the small octahedral Y site, as is also confirmed by a dominant magnetic dipolar ^5^D_0_ → ^7^F_1_-based narrow-line emission of Eu^3+^ at 598 nm in the luminescence spectra^12^. It is noteworthy that also the luminescence of Eu^3+^ can serve as an additional backup for the elucidation of local structural features, which was an established method in the 1970s – 2000s for cases when X-ray diffraction could not resolve local structural features^14^. Recording of the temperature-dependent magnetic susceptibility at lower activator fractions additionally confirms the major presence of Eu^3+^ next to traces of Eu^2+^. The combination of various techniques to elucidate the oxidation state of Eu incorporated into Rb_3_Y(PO_4_)_2_ can serve as a blueprint for other studies in phosphor research and demonstrates the necessity for careful inspection into powdered, activated samples.

Next to Eu^3+^, small amounts of Eu^2+^ give rise to a dominant violet-blue emission band with low FWHM (= 2220 cm^-1^) indicating that the Eu^2+^ ions occupy a site with a high coordination number as would be expected according to the concept of chemical pressure (see Fig. 1, top panel). Moreover, the spectral range of the emission implies a weaker ligand field splitting of the 5d orbitals, which is in line with the 12-fold coordinated Rb2 site in the structure of Rb_3_Y(PO_4_)_2_. The occupation of such a large site is uncommon for Eu^2+^ and typically only encountered if any other site in the host compound, such as the comparably small and 6-fold coordinated Y site, is even less matching. A historical, illustrative example for such an uncommon occupation is RbMgF_3_:Eu^2+^, in which the Eu^2+^ ions show rarely encountered 4f^7^ → 4f^7^-based emission in the UV range, which is only explicable by an occupation of the larger Rb sites in that perovskite-type compound^15^. The authors could assign the long tail of the emission band of Rb_3_Y(PO_4_)_2_:Eu in the visible range to the occupation of the other two available cation sites^12^, which is additionally supported by time-resolved spectroscopy. The radiative decay time of a 4f^6^5d^1^ → 4f^7^ transition of Eu^2+^ is in the order of around 1 µs. Upon increase of the emission wavelength, the radiative decay time usually gets elongated because of a lowered local photonic density of final states. This concept can be envisioned as follows: If an emitter is incorporated into a box of a fixed volume and shows luminescence, the number of standing waves with shorter wavelength per unit volume is higher than for standing waves with longer wavelengths. Thus, the chance for radiative decay per unit time is higher for emission at a shorter wavelength. Such a feature is very characteristic to 4f^*n*-1^5d^1^ → 4f^*n*^ emitters with a high internal quantum yield such as Ce^3+^ (*n* = 1) ^16^, Pr^3+^ (*n* = 2)^17^, or Eu^2+^ (*n* = 7)^18^, and was also observed by the authors of this work in *Light: Science & Applications*^12^, implying that also the other two cation sites are occupied by Eu^2+^ ions. Measurements at liquid He temperatures (4.2 K) and selective excitation and emission spectra could give more striking evidence on a powdered luminescent sample. In addition, such a temperature variation can help elucidate potential phase transitions, which is known to occur for several other aphthitalite-type compounds^19–21^.

**Fig. 1:**
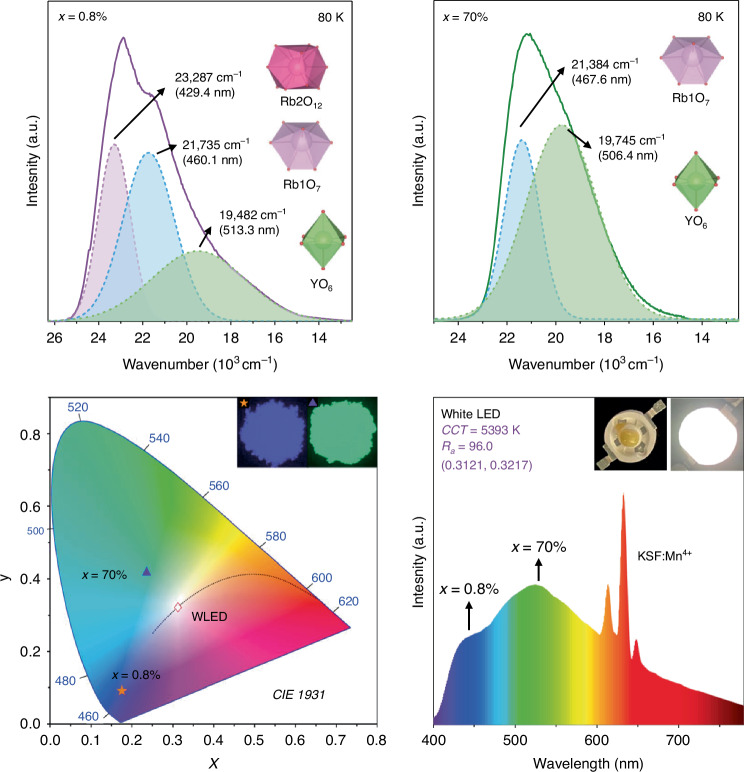
Spectra of Rb_3_Y(PO_4_)_2_:Eu for varying Eu contents and their usage in wLEDs^13^. *Top panel*: Preferential site occupation at low (0.8%) Eu concentrations. Three distinct emission bands, which cover a wide range from 400 to 600 nm, can be measured and attributed to the Rb1, Rb2, and Y sites in the sample containing 0.8% Eu. Due to the preferential site occupation in the 70% sample, only the Rb1 and Y sites are occupied which leads to two emission bands in the blue spectral range. *Bottom panel*: Manufactured prototype containing commercially available KSF:Mn^4+^ (K_2_[SiF_6_]:Mn^4+^) as well as Rb_3_Y(PO_4_)_2_:Eu with high and low Eu fractions (0.8 and 70% Eu). The corresponding CIE diagram shows excellent color rendition (*R*_a_ = 96.0, CCT = 5393 K) of the pc-wLED. The emission spectrum of the prototype shows the spectral coverage from the UV to the NIR

At larger activator fractions (>20 mol% Eu^2+^), the compromise of occupying a large site becomes noticeable and the appearance of the emission spectrum drastically changes resulting in a dominant broad green emission at room temperature (see Fig. 1, top panel). This observation is assigned to a preferential occupation of the other two available smaller cation sites, whose occupation leads to a stronger crystal field splitting of the 5d orbitals of Eu^2+^ and thus, a redshift of the luminescence. Not only does the classic empirical van Uitert relation^22^ give additional confidence in this interpretation. XANES measurements reveal that at higher Eu contents, a higher relative content of Eu^2+^ is present. Since high-energetic synchrotron radiation could potentially also affect the oxidation state of a redox-active activator, the authors employed much milder temperature-dependent magnetic susceptibility measurements as an independent proof. Investigation of magnetic properties to identify the oxidation state of an activator is a highly underestimated and powerful technique. While the 4f^6^ ion Eu^3+^ with its energetically closely separated ^7^F_*J*_ (*J* = 0…6) ground levels give rise to a Boltzmann-averaged temperature-dependent paramagnetism with a Van Vleck contribution at sufficiently low temperatures^23^, a single Eu^2+^ ion with its energetically isolated ^8^S_7/2_ (4f^7^) ground level is a textbook example of a classic spin-only Curie paramagnet resulting in a linear relation between the inverse susceptibility and temperature. DFT calculations of the formation energies of the different occupation scenarios at higher Eu contents also support the authors’ interpretation of the luminescence spectrum at 80 K.

Again, the photoluminescence of the Eu^3+^ traces offers useful information. At high nominal Eu contents, a more dominant Judd-Ofelt allowed ^5^D_0_ → ^7^F_2_ transition at around 610 nm is observable, which would formally imply the occupation of a non-centrosymmetric site. The Rb sites are very large for occupation by Eu^3+^, while the more matching Y site occupied at low Eu contents clearly gives rise to a more dominant magnetic dipolar ^5^D_0_ → ^7^F_2_ transition. X-ray powder diffraction patterns give additional insights here and demonstrate that there is phase segregation of Y_2_O_3_ at elevated Eu contents from the reaction mixture. Eu^3+^-activated Y_2_O_3_ is a well-known phosphor that can be excited efficiently at 254 nm and gives rise to strong red luminescence at 612 nm since the cubic bixbyite structure type Y_2_O_3_ crystallizes also contains (next to a *S*_6_ centrosymmetric site) a non-centrosymmetric Y site with local *C*_2_ symmetry^24,25^. The EXAFS data also reveals a Eu–O path of around 2.5 Å, which happens to match the average expected Eu–O bond length in Eu_2_O_3_. Again, it becomes evident that luminescence spectroscopy of selected emitters can become a structurally resolving tool in combination with other methods, as demonstrated by the authors of this *Light: Science & Applications* publication^12^.

Strikingly, the authors found that the incorporation of higher activator fractions is even beneficial for the photoluminescence quantum yield and leads to an increase to a value of around 40% at a nominal activator fraction of 70 mol% Eu thus increasing the overall achievable brightness by means of the enhanced absorption. This is remarkable as most phosphors usually have a much lower so-called percolation point of just a few mol%. While the quantum yield first increases linearly at very low activator fractions due to the increasing absorbance, it decreases again above a certain threshold as energy migration processes throughout the host structure may occur until the energy is released nonradiatively at a defect site leading to luminescence quenching. The percolation point depends on the explicit host structure and the interaction mechanism between the activators as was originally treated on a theoretical level by Dexter and Schulman^26^. A historical example for the immense relevance of this topic can be traced back to the 1970s when scientists tried to find an efficient gain medium for the four-level lasing activator Nd^3+^. One key concept was the usage of rare-earth-based host compounds with extraordinarily large distances between the rare-earth sites to allow for stronger absorption at higher activator fractions without the sacrifice of luminescence intensity due to concentration quenching. Successful examples include the aluminate garnets *RE*Al_5_O_12_:Nd^3+^ (*RE* = rare earth element),^27^ the huntite-type borate NdAl_3_(BO_3_)_4_^28^, the ultraphosphate NdP_5_O_14_^29^, or the metaphosphates Li*RE*(PO_3_)_4_:Nd^3+^ (*RE* = rare earth element)^30^. Nowadays, Nd:YAG is a primary standard for solid-state lasers.

The peculiar behavior of the violet-blue emitting phosphor at low Eu concentrations that turns its emission color to turquoise and green at elevated Eu^2+^ concentrations with enhanced quantum yield prompted the authors to test this effective phosphor with differing Eu content in a wLED together with the established narrow-line red-emitting phosphor K_2_[SiF_6_]:Mn^4+^ (KSF:Mn^4+^, see Fig. 1, bottom panel). With that strategy, they achieved excellent white-color rendition. The luminescence intensity of the phosphors appears to be thermally robust to up at least room temperature. It will be relevant to investigate the photoluminescence of these compounds at 150 °C, which is the typical working temperature of a pc-wLED, and put the Eu^2+^-activated phosphors to a long-time application test. The future is certainly bright – also due to the enlightening and appealingly simple approach the groups around Xia and Zhang presented in *Light: Science & Applications*^12^.
